# Supplementary material for: O group is a protective factor for COVID19 in Basque population
Source: PLoS One. 2021 Apr 7;16(4):e0249494. doi: 10.1371/journal.pone.0249494 (PMC8026022; doi:10.1371/journal.pone.0249494)
Supplement: Attachment — Submitted filename: reponse to the reviewers_ABOgroups_MMC.docx [file pone.0249494.s001.docx]

**POINT-BY-POINT RESPONSE TO THE REVIEWERS**

**Reviewer #1**

In this study, the authors compare the distributions of blood group types in (apparently) symptomatic COVID19 patients diagnosed in Gipuzkoa (Basque country, Spain) with the distributions of blood group types found in the general population, as inferred from a local Basque blood bank. They conclude that O blood group is protective, whereas A blood group is a risk factor. This study joins a handful of others, from various parts of the world, confirming an influence of blood group on vulnerability to COVID19 infection.

I have a few suggestions for the authors to consider:

1. In the abstract, I would suggest moving what is currently the second sentence (the call for replication of the work in different regions of the world) to the end of the abstract text, after the main results have been presented. I would also encourage the authors to give, in the abstract, some quantification of the degree of protection conferred by group O blood (e.g, the odds ratio).

*We thank to the reviewer for this comment. As suggested we change the abstract and add the required information.*

2. The authors state that all patients had tested positive for SARS-CoV-2 infection at Donostia University Hospital “according to the actual guidelines.” Could they please describe briefly those guidelines? Were these patients symptomatic, asymptomatic, or a mix of the two? Later, in the results, it appears that all the COVID patients had some level of clinical symptoms, but it would be good to clarify that asymptomatic individuals were excluded from the study, if that is indeed the case.
*Thanks for the comment. We added this information to the method section of the manuscript to clarify that all the COVID19 patients included in the study were symptomatic and how the positivity for the virus was tested. Approved SARS_CoV-2 qPCR test has been performed for all the samples at Microbiology department in the Donostia University Hospital. The moment in which all data for this study were retrieved, was a moment in the pandemic in which all the patients that came to the Hospital were Symptomatic.*

3. The results section states “According with the literature, in our data group A is more frequent in COVID19 patients (48.3% . . . )”; the authors need to be explicit that they are comparing this with the frequency of group A in the general Basque population.

Thanks for notice it. We have added this to the manuscript and have rewritten the sentence to avoid any misunderstanding.

4. I think the authors could add value to their paper by testing whether blood groups A, B, and AB differ from each other in terms of susceptibility to COVID infection. The authors emphasize group A, but it seems like group B might be still more susceptible, and AB perhaps the most susceptible.

We change our results section adding tests for each group.

5. The authors state that “No difference has been found in ABO group distribution with severity”, but the readers cannot evaluate this claim, because the data are not shown. I would encourage the authors to include the data broken down into severity categories, along with some formal statistical analysis of disease severity effects. This could be done in an on-line electronic supplement if the authors don’t wish to include it in the main text of the manuscript.

We add in Table 1 a distribution of the ABO groups with severity.

6. The manuscript needs a final light editing to correct numerous small problems with English grammar. (I would have called some of these out, but my copy of the manuscript does not have line numbers.)

Thanks to the reviewer for the comment. We have reviewed the manuscript and polish the language, so we hope that now the English grammar is correct.
